# Living experiences of people living with HIV-AIDS from the client’s perspective in nurse-client interaction in Indonesia: A qualitative study

**DOI:** 10.1371/journal.pone.0282049

**Published:** 2023-02-22

**Authors:** Abd Nasir, Ah Yusuf, Makhfudli, Susilo Harianto, Fanni Okviasanti, Yanis Kartini

**Affiliations:** 1 Faculty of Vocational, Airlangga University, Surabaya, Indonesia; 2 Faculty of Nursing, Airlangga University, Surabaya, Indonesia; 3 Faculty of Nursing and Midwifery Nahdlatul Ulama University Surabaya, Surabaya, Indonesia; University of Sharjah, UNITED ARAB EMIRATES

## Abstract

**Background:**

Nurse–client interaction when providing nursing services is limited to optimizing treatment and self-care, with limited focus on the psychological problems of people living with HIV-AIDS. However, psychological problems manifest more often than the health risks of the disease itself. This study aimed to determine the emotional response of people living with HIV-AIDS who received limited attention from nurses from the perspective of nurse–client relationship.

**Patients and methods:**

A phenomenological qualitative design was used through in-depth face-to-face interviews in a semi-structured manner, in an effort to obtain complete data. This research used purposive sampling with Participatory Interpretative Phenomenology analysis, involving 22 participants (14 males and 8 females).

**Results:**

This research produces several themes, with six subcategories: 1) Difficulty of social access, 2) Forcing to accept their situation and suppressing their will, 3) Wanting to be recognized like other people in general, 4) Social stigma and self-stigmatization affecting surroundings, 5) Lacking enthusiasm for life expectancy, 6) Always lingering under the shadow "when death picks up."

**Conclusion:**

The results showed that mental stress was experienced more than physical problems by people living with HIV-AIDS, thus prompting new changes to nursing services for HIV-AIDS patients that emphasize psychosocial aspects, in addition to clinical features, facilitated by satisfying relationships between nurses and clients to provide quality services.

## Introduction

The complex issues faced by people with HIV-AIDS prioritizes the provision of comprehensive nursing care [1]. In Indonesia, the number of people living with HIV-AIDS remains relatively high, with a Case Fatality Rate of 1.03% as of 2018 [2], and is often accompanied by comorbidities, ranging from infectious diseases such as pulmonary TB, accounting for 21.65% and non-communicable diseases, such as hypertension (39.17%) [3], and Kaposi’s sarcoma (2.6%) [4]. This is the main cause of hospitalization in patients [3], with a prevalence of 59% of the population dominated by individuals in productive age. In addition, the cost of care provided to these patients was almost six times higher than that of the controls and four times higher after taking antiretroviral drugs [5].

HIV-AIDS, which is characterized by a progressive decline in the immune system, has a serious impact on well-being. This is evidenced by the fact that patients with other diseases, such as pulmonary TB, are still willing to be open to appearing in public [6]; however, people with HIV-AIDS are very protective in their expression [7]. This difference is driven by the general stigma attached to people with HIV-AIDS in the community, which is far more discriminatory than other diseases [8], and the belief that HIV-AIDS is a punishment from God and the sins caused by their actions can add to the burden of suffering [9]. Therefore, it is important to understand the emotional responses of people living with HIV-AIDS during therapy programs.

The severity experienced by people living with HIV-AIDS does not correspond with clinical practice because nurses fail to respond adequately to their demands, thus affecting their overall well-being [10]. This discrepancy between expectations and reality experienced by these patients, as well as worsening health as a result of their illness, emerged as a very agonizing experience, as reported in this study [11]. Meanwhile, the dominance of optimizing the anticipation of loss to follow-up and strengthening self-care has eliminated problems from other psychosocial aspects, so that it can change the healthcare system, which includes bio, psycho, social, cultural, and spiritual aspects [12].

It is clear that attention to physical problems is a serious concern without considering psychosocial problems or traumatic experiences when people living with HIV-AIDS interact in the community. For a wider interest in nursing practice, provision of nursing care to people living with HIV-AIDS should focus on psychosocial problems besides the physical aspects within the framework of holistic nursing care [13]. This is because, while providing nursing care to people with HIV-AIDS, the approach taken is concentrated and centered on clinical practice [14].

Meanwhile, the “Health” paradigm toward HIV-AIDS has shifted to a view of disease as a result of human behavior, and this must be integrated into the beliefs of people living with HIV-AIDS to support their well-being, which requires knowledge and an understanding of the patient’s personal experiences and the meaning of these experiences in a patient’s life, as these are much more important than just clinical pharmacological monitoring [15]. There is a relationship between well-being and increased immunity [13]. Therefore, in order to improve quality of life and understand their life experiences, it is possible to develop nursing interventions that can improve their well-being [16].

People living with HIV-AIDS frequently experience stigma and discrimination, especially in the surrounding community. This triggers psychological problems, including anxiety, depression, and the risk of suicide [17]. In this approach, the focus is on the experiences of people living with HIV-AIDS, and nursing care is critical because nurses as caregivers play an essential role in every nursing action and follow-up in continuing nursing services [18]. Therefore, nurses must view people living with HIV-AIDS as a combination of bio, psycho, socio-cultural, and spiritual features, without distinguishing ethnicity, nationality, race, and religion. The relationship between patients and nurses is professional [12]. It is important for nurses to develop nursing care by enhancing interpersonal relationships between them and the patients as a mutually beneficial relationship; this approach encourages people-centered holistic nursing practice [19].

In addition, in the process of caring, the depth of the patient’s experience is easy to understand, and nurses obtain new possibilities and opportunities to provide nursing services according to the needs and expectations of the patient [20]. Therefore, understanding the feelings of people living with HIV-AIDS is important, and this is used for the construction of future nursing interventions to improve patients’ health status and happiness. Although several qualitative studies have reported events experienced by people suffering from HIV-AIDS [21], they did not specify the relationship between nurses and clients from the client’s perspective and only focused on physical and functional disorders. Therefore, this study focuses on studies on the life experiences of people living with HIV-AIDS when interacting with nurses, and the meaning of these experiences.

## Material and methods

### Participants

Overall, the study involved 22 participants, and factors included age, gender, type of work, and comorbidities. Further details can be found in Table 1.

**Table 1:** Participants’ demographic profiles.

Individual Characteristics	Freq	Percentage (%)
Gender		
Male	14	0,64
Female	8	0,36
Marital Status		
Single	9	0,41
Married/live together	10	0,45
Divorced/widowed/widow	3	0,14
Level of education		
Primary school	6	0,27
First Middle School	3	0,14
Senior High School	10	0,45
Diploma/ College	3	0,14
Work		
Homeworker	8	0,36
Unemployment	1	0,05
Informal employee	7	0,32
Formal employee	6	0,27
Monthly income		
500.001–1.000.000	6	0,27
1.000.001–2.000.000	4	0,18
2.000.001–3.000.000	2	0,10
≥ 3.000.000	10	0,45
Sexual Orientation		
Homosexual	9	0,40
Heterosexual	11	0,50
Bisexual	2	0,10
Residential Arrangement		
Living alone	3	0,14
Living with other people	19	0,86
Transmission / At-risk lifestyle		
Injecting Drugs	1	0,05
Sexual	21	0,95
Smoke		
Yes	7	0,32
No	15	0,68
Alcohol Consumption		
Yes	1	0,04
No	21	0,94
Economic Environment		
Enough	20	0,91
Good	2	0,09

### Procedure

The life experiences of people living with HIV-AIDS that are overlooked by nurses during the interaction process as meaningful experiences have been mutually agreed upon in this study. In addition, the researchers used the COREQ strategy to determine consolidation criteria in conducting qualitative searches for this study [22]. The study process followed the Consolidated Criteria for Qualitative Research Reporting checklist. Individual and in-depth face-to-face interviews with people living with HIV-AIDS were conducted in a semi-structured manner to obtain complete data on life experiences when interacting with nurses [23].

In order to meet the participants, mediation was obtained from nurses who organized the HIV-AIDS Disease Prevention and Eradication program at the Health Office, and were placed in the Community Health Center to monitor and provide treatment and counseling services. The nurse assessed whether the participants (people living with HIV-AIDS) were ready to be interviewed, initially asking potential participants if they agreed to be approached by the researcher to be invited to participate in the study. The researcher offered the participant information sheet to be read to the participants; if they were willing to participate, they provided written consent. Participants were encouraged to reflect on generally-accepted healthcare services and discuss their situations when interacting with nurses. Interview guides were used to remind researchers of the topics covered and ensure that all major topics were covered, including discussions of life experiences during their interactions. The participants were interviewed in a separate quiet room with treatment services that had been prepared in advance at the Community Health Centers. They had the option to stop the interview at any stage, if they wished. The results of individual interviews were recorded using cell phones, carefully written, and confronted with nonverbal responses through field notes for data analysis, and then reviewed to improve data accuracy [23].

### Study design

A phenomenological qualitative approach was used in this study [24]. The sample was selected through purposive sampling of patients with HIV-AIDS. To gain meaningful perceptions and experiences, the researchers recruited a diverse and representative sample that reflected the population of people living with HIV-AIDS. The inclusion criteria in this study were as follows: (1) people living with HIV-AIDS who were hospitalized or outpatients, (2) people living with HIV-AIDS who have had HIV-AIDS for more than two years, and (3) people living with HIV-AIDS routinely seeking treatment. Meanwhile, the exclusion criteria were people living with HIV-AIDS who were seriously ill; therefore, interviews were not possible. Data saturation was the final limit for determining the number of samples. Data saturation was reached at the 21^st^ interview because no new information emerged [23]. To strengthen the data, an additional participant was interviewed to ensure that no new information was obtained. Therefore, for the 22^st^ participant, data saturation was achieved. Furthermore, the researchers conducted a thematic analysis using a deductive method approach [23] by exploring important themes that described the phenomena that occurred in people living with HIV-AIDS when interacting with nurses.

### Data analysis

All interview recordings were rewritten verbatim, coded, and labeled, and transcription of all data was carried out to be consistent with data reflection activities and used to determine new ideas. Interpretative Phenomenological Analysis was used for data analysis [25]. Researchers have also developed an Interview Guide (Box 1) based on the research objectives and the existing literature. Interview transcripts and field notes were read carefully and repeatedly to determine emerging themes by reading sentences in detail and then categorizing important terms that were related to each other through a selective approach.

**Box 1. Interview guide.**

The interview guide was developed for individual interviews based on a literature review. The interview guide was piloted with two HIV-AIDS sufferers. The content of the guide was relevant with no amendments required and included open-ended questions such as:

1. What has changed since you suffered from HIV-AIDS?
2. What do you want, now and in the future while living with HIV/AIDS
3. What is difficult while living with HIV/AIDS, and what do you think about while living with HIV/AIDS
4. How did you feel during the treatment program at the health service? What do you want now but cannot be fulfilled by nurses in health services?
5. How did this affect you?
6. Is there anything very worrying about your situation while undergoing the treatment program at this health service?

The researchers read the text collectively, tried to understand the overall meaning, and developed keywords and concepts through dialogue with the text. Additionally, the researchers maintained openness by reflecting on various interpretations to monitor assumptions and biases through the triangulation process, namely, linking interview data with field notes to clarify what is meant by clarifying to participants [26].

Each sentence was analyzed by the researchers and confronted with data in the field notes. These themes were then reconstructed into a description of the life experiences of people living with HIV-AIDS that nurses overlooked when interacting [26], The researchers then connected categories based on the events experienced, and always paid attention to the balance of research themes by looking at each part as a whole.

### Ethical considerations

The research procedure was performed in accordance with the principles of the Declaration of Helsinki and was approved by the Research Ethics Committee of the University of Muhammadiyah Lamongan (number:085/EC/KEPK-S2/05/2021. All participants provided informed consent and were told that they could withdraw from the study at any time. Informed consent was obtained from each study participant for publication of their responses while maintaining anonymity, and the place and time for the interview were arranged to maintain privacy and confidentiality. The respondents’ identities were anonymized to maintain confidentiality.

## Results

Broadly speaking, the theme that emerged was that people living with HIV-AIDS received limited attention regarding their psychosocial problems when interacting with nurses. Six sub-themes were found to support the overall theme:1) Difficulty in social access, 2) Forced to accept their situation and suppress their will, 3) Wanting to be recognized as others in general, and 4) Social stigma and self-stigmatization that affects the surroundings, 5) lack of enthusiasm for life expectancy, 6) always imagining being in the shadow "when death picks up.".

### The difficulty of social access

HIV-AIDS affects all personal aspects of people infected, including social access, which makes them disconnected and lose their future. As a participant said:

“I used to work, make friends, and hang out with other friends and family like normal people before I got this disease (HIV-AIDS) … but right now it’s really hard for me to do these things. I feel forced to concentrate on a strict routine of treatment and self-care programs for my disease.”

(P-16).

This situation causes people living with HIV-AIDS to lose control of their lives, lose enthusiasm, become disappointed, and regretful. According to a woman:

“My condition prevented me from doing what I wanted. There’s nothing I’m proud of myself right now, so I’m so sad … I’m so traumatized that I have to erase all my dreams.

(P-03).

### Forcing to accept their situation and suppressing the will

Participants often cited self-acceptance as a very positive attitude in their lives, and they were able to calm down even though they realized that it would take some time. One of the participants said:

“I have to accept my condition. I don’t force myself to do things that are difficult for me to do … I try not to be confused and not to worry, because I have to condition this situation."

(P-10),

The situation experienced by HIV-AIDS patients forces them to accept their situation, even though there is a desire to rebel.

“I let my illness damage my body, I accept it even though I have to lose my beauty … it’s already done… unless I surrender to God, maybe this is a way of life that I have to accept”

(P-09).

### Wanting to be recognized like other people in general

The perception of “pressure-free” in daily life makes participants feel that their lives are more relaxed, comfortable, and free, and their emotions more controlled. All desires can be carried out, such as getting along with everyone, and everyone can see them as normal people on an equal footing. Similar to what this lady said:

“I want to be like him (a volunteer HIV-AIDS assistant who is also an HIV-AIDS patient) … it turns out that he can be like normal people in general … he is very relaxed. Having this disease (HIV-AIDS) can lead to a life where you do not have this disease. Finally, I believe that I can live a life like others because what he can do I can also do, even though I have to struggle.”

(P-08).

They wanted others to treat their illness like any other infectious disease. They also did not want to be seen as “different” from their surroundings. Another participant pleaded:

“I realized that I had this disease (HIV-AIDS) … but I didn’t want the Health Officers to ask me what disease it was … he should have known what disease I had. He looked like he didn’t know and didn’t want to know my feelings. And should I answer that question (about HIV-AIDS) when many people ask me?”

(P-05).

### Social stigma and self-stigmatization can affect surroundings

This theme shows that people living with HIV-AIDS are aware that they are limiting their family life. Concurrently, they feel the need for their family. Participants believed that their condition had a negative impact on others. They believed that they were limiting their family life and that they were a burden to their family, but also desperately needed constant attention. One of the men expressed his feelings:

“I feel very sorry for you (wife), because you carry a very heavy burden … I am being treated; you are also willing to wait for days when I have to go to the hospital, take me to control treatment at the hospital, and always accompany me … Activities in the surrounding community, as well as daily work, are always left to you, and makes it difficult for you”

(P-06).

This makes people living with HIV-AIDS feel tired, unmotivated, and hopeless. To avoid bothering other family members, they did not want to express their grievances. However, they felt ambiguous between wanting to be helped because they were still weak and needed help, and a desire not to bother their families. Some patients said that family support was important, but they felt frustrated that the support provided was useless and only inconvenienced their family.

“My brother plays a significant role in my life; he watches me all day and helps me with everything. Without him, I don’t know what will happen to me. However, I know that he only thinks about me and doesn’t care about my needs. I feel bad … I am very sad because it always bothers my family, even though the effort is not worth the results obtained … until now, I have not shown any significant changes.”

(P-15).

### Lacking enthusiasm for life expectancy

Having hope is the main weapon for people living with HIV-AIDS to survive and fight the disease, especially when the disease begins to progress. This hope helps them optimistically look to the future; however, it is that they never get. One mother said:

“I always hope that I could live this life well, that this life deserved to be enjoyed… but my body is always controlled by this disease, it seems there is nothing to wait for (HIV-AIDS)’

(P-02).

The participants hoped that their illness would improve, but when they waited for hope, there was only despair, because what they wanted was never achieved, but when they never hoped, they wanted to get better quickly, as shown by the following quote:

“The support of the people closest to me has helped me a lot … however, when they are beside me, I feel that the support cannot change my condition … I am still like this and always filled with suffering”

(P-07)

### Always imagining the shadow "when death picks up"

This theme describes the recurring thoughts of the patient about death. HIV-AIDS is frightening, as if death is in sight. Meanwhile, on other occasions, they are resigned and ready to face death. Some participants expressed that death was coming soon. A man expressed his feelings in the following way:

“I know that the medicine I take is only to survive … only a few people survive, and in the end they also face death. Especially when I experience chills, I’m so scared, and I always think that my end has come”

(P-18)

Other participants also revealed:

“I just surrender, and I’m ready to be picked up by death at any time”

(P-17)

Meanwhile, there were participants who wanted to die. In this case, death is seen as a solution to end their suffering, so there are those who think about planning suicide. One teenager confessed the following:

“I am very tormented by this disease (HIV-AIDS), I don’t think I can stand this suffering, and at some point in the day, I even think about ending my life, so I don’t have to endure this suffering for too long.”

(P-21)

## Discussion

Overall, this study emphasizes the physical impact of HIV-AIDS that affects feelings, purpose in life, and relationship with their immediate environment and social life, and several previous studies have reported social restrictions due to the negative impact of HIV-AIDS [27]. Uncertainty about their health condition is an additional feature, as a result of suffering from HIV-AIDS, as in this study [28]. However, previous studies have focused on behavioral follow-up of strict treatment regimens [29], medication adherence and routine control behaviors [30], and others have reported beneficial effects [31]. Despite these benefits, they are forced to make changes from routine activities that must be undertaken into new patterns of social life, and this requires major changes and readjustment into a personal life that can cause considerable discomfort to them. Regarding events experienced by people living with HIV-AIDS that make sufferers feel depressed, nurses must be physically present to discuss what to do, examine previous experiences, and help them assess and decide how to maintain their lifestyle to meet the self-care needs of people living with HIV-AIDS and/or modify their physical, psychological, and social environments according to their current situation, conditions, and demands [32].

Furthermore, other studies have also reported that having HIV-AIDS can lead people to see a change in their identity, and ultimately, they are able to judge and realize that they will never be who they were before [33]. However, this study has reported that this perception has a very negative impact on nurses, leading to prolonged sadness, stress, low motivation, and withdrawal, and it is increasingly difficult for nurses to understand if they are introverted and unwilling to express their feelings. This situation is a major problem that must be considered, and several researchers have reported positive consequences of efforts to increase self-esteem [34]. This is an important study by nurses to build effective communication that focuses on problems related to their ideas, thoughts, feelings, and hopes, through which nurses can contribute to altering their self-concept through changes in the construction of new identities [35].

The findings that nurses should be wary of are that participants conceal their problems and are unwilling to open up with nurses, even with their families, and they secretly make plans beyond common sense without the family’s knowledge. This behavior is detrimental to them because they do not have time to share their experiences and stop their routine activities. However, this is not the case with the results reported in studies related to other chronic diseases where patients always express their feelings [36]. Family members had a positive influence on the disease process analyzed, especially those related to daily activities and psycho-emotional support. Other studies also support this finding, which explores the perspective of families in supporting people living with HIV-AIDS regarding their quality of life related to the burden borne [37]; many families are forced to change their lifestyle to provide support to those they care about [38]. Therefore, it is important for families to learn patterns of providing support and combining meaning in life for the benefits provided.

This study also highlights that people living with HIV-AIDS have recurring thoughts about death. Another study also reported the same situation, especially when it came to the vital threat felt by patients with terminal cases so that they had difficulty expressing their feelings [39]. The results of this study highlight the concerns and suffering as part of their life experiences, especially those who are extremely scared of the possibility of dying, and some patients like this, as reported in this study [40], are not prepared for the possibility of death. However, despite the results of this study, qualitative studies have reported that some patients face this process calmly, there is no burden in their lives, and they see their illness as part of a life cycle that must be accepted [41] because all humans return to God. The results of this study also showed that some participants had a desire to end their lives and might view death as the best way to end their illness, as reported in this study [42]. Furthermore, this study also found a desire for a patient to end his life to end his suffering. Regarding the consequences of this problem, other studies have also reported that the roles of family and health workers worsen their psychological condition because they seem to let these patients die as if they avoid their duties and responsibilities [39]. However, there is a glimmer of hope for a terminal patient, such as a person living with HIV-AIDS, to enjoy his life, and if he has to die because of his condition, the hope that arises is to die peacefully and happily. In all these circumstances, it is important for nurses to establish effective communication with patients in a “physically present” effort, which is a starting point for understanding the possibility of death [43]. In the context of interactions in nursing, patients feel that the nurse is always there. This meeting makes them willing to discuss their panic so that it can increase prosperity and peace [44], as patients and their families wish nurses to be present in their situation.

A strong desire to be able to enjoy life as a person who does not have a burden and hopes that the environment also accepts people with HIV-AIDS is an interesting finding to be discussed in this study. This finding is relevant to the goals and expectations of a prosperous life in their environment that they have experienced before suffering their illness. This is obtained when the environment around them does not make them feel depressed, so that they can live without psychological burdens. However, there have not been many reports in other studies that have been found to campaign for “free of stigma and discrimination” for people living with HIV-AIDS. However, several studies on other chronic diseases have discussed the importance of an atmosphere free from stigma and discrimination, and to this end, have discussed the importance of a stress-free life as a key to the psychological well-being of many of these patients [34]

Several studies have acknowledged that people with chronic diseases need to recover their perception of a state without mental stress and adapt to a new environment [45]. In line with this, researchers have also found that people living with HIV-AIDS want to be identified as people suffering from the disease in general and there is no “emphasis of the HIV-AIDS accent word” for their disease in their environment. In this case, the stigma experienced, both social stigma and self-stigmatization, by people living with HIV-AIDS, is also experienced by those suffering from other chronic diseases [36]; therefore, as the findings of this study show, some people in their environment try to isolate them. In this case, as also reported in this study [46], living with an HIV-AIDS person is felt very differently and is considered psychologically stressful, and these feelings can lower self-esteem and increase insecurity.

Another interesting finding of this study is their expectations. The participants agreed that there was a desire to live like they did before the illness, and this was to improve mood, quality of life, and a prosperous life [47]. On the contrary, as the researchers have seen in this study, when hope is far from the mind, despair and sadness become part of one’s life, which is in line with this study [48]. This negative emotional response has been discussed in depth among people living with HIV-AIDS. In addition, this study also found that there was no hope for patients in crisis and unstable situations, thus giving the impression of being helpless in their fight against the illness. Studies conducted on other populations show that hope is an important mediator of effective coping strategies, although this is very difficult to be shown by patients with terminal cases [49].

It is important for nurses to find the relevance between expectations and problem-solving strategies faced by people living with HIV-AIDS, that the presence of high expectations from people living with HIV-AIDS allows nurses to motivate patients to decide on appropriate actions and overcome obstacles, including their experiences as a result of the disease. In this context, the nurse plays the role of a motivator and initiator in seeking a meaningful future in their lives [50].

## Limitations

The participants in this study were comfortable expressing their experiences while interacting with nurses during the ARV treatment program, but with different characteristics and emotional responses, their perceptions may also be different. They were very careful about expressing their feelings. In addition, the way they expressed their feelings also differed. In addition, they also did not want to be open to expressing all their wishes and hopes for the services provided by nurses because the focus of service was centered on definitive treatment. Although both pose potential limitations, they are not a barrier to obtaining natural and complete data. Armed with a therapeutic communication approach, all physical, psychological, and social problems can be expressed comprehensively. The analysis is based on the data that have been found, but triangulation with experts and HIV-AIDS program holders may contribute much more to their knowledge and perceptions through a professional approach, including with health care workers.

## Conclusion

The interaction framework between nurses and clients in this study has made it possible to gain a greater knowledge and understanding of the meaning of the life experiences of people living with HIV-AIDS and to encourage new changes in providing nursing care for these patients. Furthermore, nurses innovate and reshape the nursing service system, which is centered on an individual approach. Therefore, nurses can position themselves optimally to become familiar with the patient’s experience, and emphasize and maintain interpersonal relationships with patients and their families. This strategy will result in better nursing services and greater satisfaction for patients and professionals who care for them.

## Supporting information

S1 File (DOCX)
