# Supplementary material for: Living experiences of people living with HIV-AIDS from the client’s perspective in nurse-client interaction in Indonesia: A qualitative study
Source: PLoS One. 2023 Feb 22;18(2):e0282049. doi: 10.1371/journal.pone.0282049 (PMC9946244; doi:10.1371/journal.pone.0282049)
Supplement: S1 File — (DOCX) [file pone.0282049.s001.docx]

VERBATIM

**The difficulty of social access**

HIV-AIDS disease affects all personal aspects of sufferers, and all of this has an impact on social access that makes them disconnected and lose their future. As participants said:

“I work, make friends, and hang out with other friends and family like normal people before I got this disease (HIV-AIDS) … but right now it's really hard for me to do, I feel forced to concentrate on going through a strict routine of treatment and self-care programs for my disease” (P-16).

This situation causes people living with HIV-AIDS to lose control of their lives, lose enthusiasm, become disappointed, and can only mourn. A woman expressed this feeling as follows:

“My condition prevented me from doing what I wanted. There's nothing I'm proud of myself right now, so I'm so sad … I'm so traumatized that I have to erase all my dreams” (P-03).

**Forcing to accept their situation and suppressing the will**

Participants often cited self-acceptance as a very positive attitude in their lives, and they were able to calm down, even though they realized that it would take some time. One participant said:

“I have to accept my condition. I don't force myself to do things that are difficult for me to do … I try not to be confused and not to worry, because I have to condition this situation " (P-10),

The situation experienced by HIV-AIDS requires them to agree to what is happening to themselves, even though there is a desire to rebel.

“I let my illness damage my body, I accept it even though I have to lose my beauty … it's already done… unless I surrender to God, maybe this is a way of life that I have to accept” (P-09)

**Wanting to be recognized like other people in general**

The perception of “pressure-free” in daily life makes participants feel that their lives are more relaxed, comfortable, free, and their emotions are more controlled. All desires can be carried out such as getting along with everyone, and everyone can see them as normal people on an equal footing with others. Like what this lady said:

“I want to be like him (a volunteer HIV-AIDS assistant who is also an people living with HIV-AIDS) … it turns out that he can be like normal people in general … he is very relaxed. It turns out that having this disease (HIV-AIDS) can lead a life like not having this disease (HIV-AIDS). Finally, I believe that I can live a life that can be done by others because what he can do I can also do, even though I have to go through struggles.” (P-08).

They wanted others to treat their illness like any other infectious disease. They also wanted not to be seen as “different” among their surroundings, another participant pleaded:

“I realized that I had this disease (HIV-AIDS) … but I didn't want the Health Officers to ask me what disease it was … he should have known what disease I had. He looked like he didn't know and didn't want to know about my feelings. And should I answer that question (about HIV-AIDS) when many people ask me?” (P-05)

**Social Stigma and Self-Stigmatization Affecting His Surroundings**

This theme shows that people living with HIV-AIDS are aware that they are limiting their family life; However, at the same time, they feel the need for their family. Participants believed that their condition had a negative impact on others. They believed that they were limiting their family life and that they were a burden to their family, but also, desperately they needed their constant attention. A man expressed his feelings:

“I feel very sorry for you (wife), because you carry a very heavy burden … I am often treated, you are also willing to wait for days when I have to go to the hospital, take me to control treatment at the hospital and always accompany me … Activities in the surrounding community, as well as daily work, are left only for a person who always make it difficult for you” (P-06).

This condition makes people living with HIV-AIDS feel tired, unmotivated, and hopeless. In order not to be a bother to other family members, they did not want to express their grievances. However, they felt ambiguous between wanting to be helped because they were still weak and needed help and a desire not to bother their family. Some patients said that family support is important, but they felt frustrated that the support provided was felt useless and only inconvenienced their family.

“My brother is a significant person in my life; he watched over me all day and helped me with everything. Without him, I don't know what will happen to me. However, I know that he only thinks about me and doesn't care about their needs. I feel bad … I am very sad because it always bothers my family, even though the effort is not worth the results obtained … until now, I have not shown any significant changes.” (P-15).

**Lacking enthusiasm for life expectancy**

Having hope for people living with HIV-AIDS is the main weapon to survive and fight the disease, especially when the risk of disease begins to attack and the severity of the disease begins to share with the disease. This hope helps them optimistically to look to the future, however, this is that they never get. One mother said:

“I always have hope that I could live this life well, that this life deserved to be enjoyed… but my body is always controlled by this disease, it seems there is nothing I could wait for (HIV-AIDS)” (P-02).

The participants hoped that their illness would improve, but when they waited for hope, there was only despair, because what they wanted was never achieved, but when they never hoped, they wanted their illness to get better quickly, as shown by the following quote:

“The support of the people closest to me has helped me a lot … however, when they are beside me, I feel that the support cannot change my condition … I am still like this and always filled with suffering” (P-07)

**Always imagining the shadow "when death picks up"**

This theme describes the recurring thoughts that the patient has about the death. HIV-AIDS poses a frightening threat, as if death is in sight, meanwhile, on other occasions, they are resigned and ready to face death. Some of the participants expressed that death is coming soon. A man expressed his feelings in this way:

“I know that the medicine I take is only to survive … only a few people survive, and in the end they also face death. Especially when my body feels chills, I'm so scared, and I always think that my end has come” (P-18)

Other participants also revealed:

“I just surrender, and I'm ready to be picked up by death at any time” (P-17)

Meanwhile, because of the people living with HIV-AIDS, there are participants who want to die. In this case, death is seen as a solution to end the suffering experienced, so there are those who think about planning suicide. One teenager confessed as follows:

“I am very tormented by this disease (HIV-AIDS), I don't think I can stand this suffering, and at some point in the day, I ever think about ending my life so I don't have to endure this suffering for too long.” (P-21)
